# Supplementary figures and images for: CD4+ and CD8+ cell counts are significantly correlated with absolute lymphocyte count in hospitalized COVID-19 patients: a retrospective study
Source: PeerJ. 2023 Jun 23;11:e15509. doi: 10.7717/peerj.15509 (PMC10292192; doi:10.7717/peerj.15509)

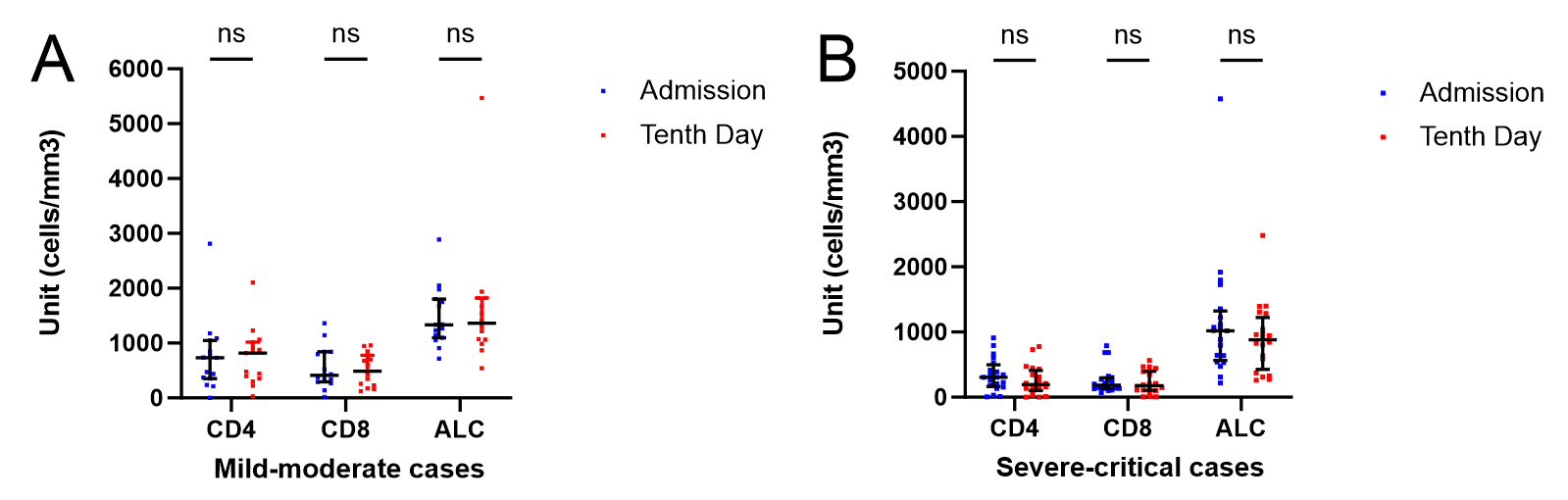

Supplement: Supplemental Information 1 — (A) Mild-moderate patients, (B) Severe-critical patients [file peerj-11-15509-s001.png]
